# Supplementary material for: A theory on individual characteristics of successful coding challenge solvers
Source: PeerJ Comput Sci. 2019 Feb 4;5:e173. doi: 10.7717/peerj-cs.173 (PMC7924443; doi:10.7717/peerj-cs.173)
Supplement: Supplemental Information 2 [file peerj-cs-05-173-s002.rtf]

Instructions for Participants to “A theory on individual characteristics of successful coding challenge solvers”
Note: this is an English translation of the instructions we provided the participants of the study.
<after reading the consent form>
Do you have questions regarding the consent form? It is important to understand that I will be able to see from whom the answers to the questionnaires and the solutions to the coding challenges are. These data will be anonymized shortly after you complete the study.
Do you study software engineering?
Are you familiar with Eclipse and Java?
There will be three coding challenges in total and between them you have to fill out questionnaires. At the end of each questionnaire there is the instruction to raise your hand. I will then open the next coding challenge for you.
For each coding challenge a method signature is given. It is not allowed to change this signature, so that the type of the return value and the parameters of the methods are well-defined. Above the method signature, there is a comment explaining what the task is.
It is allowed to create private methods if this helps you to structure your solution or to solve the problem.
It is not allowed to use the Internet when solving the coding challenges. For answering the questionnaires it is allowed to use the Internet. You will be asked, for example, for your DSA mark. If you do not remember it, it is fine to look it up on the Internet.
It is allowed to use all methods and data structures from the standard Java package (import java.∗).
It is allowed to run the code. With each challenge there is also a main method given which contains a call of the method that you have to implement. For the input of that call the expected output is given as well. You are allowed to modify the main method if desired.
I provide you pen and a paper for taking notes.
For each challenge there is a time limit for understanding the problem, finding an algorithm and implementing the algorithm. For example, for the first coding challenge this time limit is 15 minutes. If you want to submit a solution before the time is up, please tell me and we will continue. This will not affect the score for your solution, so it might be a good idea to use the remaining time in order to improve your algorithm.
We evaluate your solutions for correctness and time complexity. It is always better to have a correct solution than no solution. And the more efficient the solution is in terms of the Big O notation, the better the score.
Please do not use your mobile phone in the next 90 minutes.
Do you have any questions?
